# Performance of custom made videolaryngoscope for endotracheal intubation: A systematic review

**DOI:** 10.1371/journal.pone.0261863

**Published:** 2022-01-06

**Authors:** Pawan Kumar Hamal, Rupesh Kumar Yadav, Pragya Malla

**Affiliations:** 1 Department of Anaesthesiology and Intensive Care, National Trauma Center, National Academy of Medical Sciences, Mahankal, Kathmandu, Nepal; 2 Department of Biochemistry, Nepal Medical College and Teaching Hospital, Kathmandu, Nepal; Illawarra Shoalhaven Local Health District, AUSTRALIA

## Abstract

**Introduction:**

Videolaryngoscope is regarded as the standard of care for airway management in well-resourced setups however the technology is largely inaccessible and costly in middle and low-income countries. An improvised and cost-effective form of customized videolaryngoscope was proposed and studied for patient care in underprivileged areas however there were no distinct conclusions on its performances.

**Method:**

The study follows PRISMA guidelines for systematic review and the protocol in International Prospective Register for Systematic Reviews. The primary aim was to assess the first attempt success of customized videolaryngoscope for endotracheal intubation. The secondary objective was to evaluate the number of attempts, laryngoscopic view in terms of Cormack Lehane score and Percentage of glottic opening, use of external laryngeal maneuver and stylet and, the airway injuries after the endotracheal intubation.

**Result:**

Five studies were analyzed for risk of bias using the National Institute of Health Quality Assessment Tool for cross-sectional studies. Most of the studies had a poor to a fair level of evidence with only one study with a good level of evidence. Certainty of evidence was “very low” for all eligible studies when graded using the Grading of Recommendation, Assessment, Development and Evaluation approach for systematic review.

**Conclusions:**

The certainty of the evidence regarding performance of custom-made videolaryngoscope compared to conventional laryngoscope was very low and the study was performed in small numbers with fair to the poor risk of bias. It was difficult to establish and do further analysis regarding whether the customized form of videolaryngoscope will improve the first attempt success rate for tracheal intubation, reduce the number of attempts, improve the laryngoscopic view, require fewer external aids and reduce the incidences of airway injury with the given low-grade evidence. Some properly conducted randomised clinical trials will be required to further analyze the outcome and make the strong recommendations.

## Introduction

Videolaryngoscope is regarded as the standard of care for airway management in places with adequate resources [1]. Videolaryngoscope when compared to traditional laryngoscope can facilitate better glottic view, reduce the number of attempts for tracheal intubations in patients with difficult airways and reduce the laryngeal trauma [2]. However, this technology is largely inaccessible in middle and low-income countries where the availability of resources is always a problem mainly related to the cost of procuring the equipment [3–5]. Many modifications have been proposed in resources constrained countries with a reduced cost and use of locally available materials. The modifications were done with the purpose of teaching and learning [6] and patient care, mainly at the time of health crisis [3]. However, it is yet to be determined whether such modification achieved the desired level of performance in terms of improving the success of endotracheal intubation, laryngoscopic view and reducing a number of attempts and the airway injuries thus ensuring the safety of the patients. Previous systematic reviews have analyzed mainly the standard form of the videolaryngoscope with specific modifications, but do not take into consideration the performance of improvised form of these videolaryngoscope which are being used in a crisis situation and in places where resources are scare. Our primary aim was to assess the first attempt success of endotracheal intubation using a customized form of videolaryngoscope. Our secondary aim was to assess the performance of medical personnel in terms of the number of attempts required for successful endotracheal intubation, improvement in laryngoscopic view, use of external aids, and the airway injury after the use of modified form of videolaryngoscope.

## Methods

### Protocol

The manuscript was prepared according to the PRISMA statement for systematic reviews (S1 Table) and meta-analysis [7] and the Plos One journal guidelines. The protocol was also registered and published in International Prospective Register for Systematic Review (PROSPERO) with the Registration number CRD42021259143.

### Information source

We searched databases of MEDLINE (Medical Literature Analysis and Retrieval System Online), EMBASE (Excerpta Medica dataBASE), Scopus from 2011, May 31 to 2021, May 31. We also searched the clinical trial registry database (clinicaltrials.gov, assessed on July 15, 2021) [8] during the same period time. The Boolean search strategy was used using the different terms to find the population (anesthesiology, anesthesiology, medical intern, medical resident), intervention (videolaryngoscope, video-laryngoscope, custom, cost, affordable, inexpensive) and, outcome (first-pass success, number of attempts, intubation time, Cormack Lehane grade, Percentage of glottic opening, external laryngeal maneuver, stylet use, airway injury). The details of search terms, timeline, and language are outlined in S2 Table.

### Eligibility criteria

Studies that included the use of any form of customized videolaryngoscope for intervention purposes in patients were included in the study. Randomised controlled trials, non-randomised studies, observational studies with the intervention were included. Studies, where intervention was done in manikin for purpose of teaching and learning, were excluded from the study. Editorials, views, or the study where the customization of the videolaryngoscope was proposed without any intervention were also excluded.

### Data collection and analysis

Two authors (PKH, PM) independently searched using the search terms in different databases. Duplicates in the search engines were removed. The remaining studies were screened for title and abstract to remove irrelevant studies not fitting the inclusion criteria. Full-text searches of relevant titles and abstracts were then searched. In case of disparities during the process, it was resolved by discussion between all three authors and the final decision was made based upon the majority’s decision. Study characteristics and outcomes were independently extracted from the eligible studies by the two investigators out of three. Only studies which were published in English during the study duration were taken in consideration from the start of the search for the following outcomes.

### Primary outcomes

The first attempt success of endotracheal intubation was taken as the primary outcome. It was defined as the passage of the tracheal tube through the vocal cord and confirmed by direct visualization or capnography or chest auscultation.

### Secondary outcomes

1. Intubation time: Intubation time was taken from the insertion of laryngoscope into the oral cavity to the insertion of the tracheal tube through the vocal cord or confirmed by capnography.
2. The number of attempts of endotracheal intubation: This was taken as the number of attempts required for successful endotracheal intubation.
3. Laryngoscopic view: Percentage of glottic opening (POGO) score and Cormack Lehane grading (CL) was taken for laryngoscopic view at the time endotracheal intubation.
4. External laryngeal manipulation: It was taken as any form of manipulation of the larynx externally to aid in the visualization and insertion of the tracheal tube.
5. Stylet use: It was taken as any form of a stylet that was used to aid in process of endotracheal intubation.
6. Airway injury: It was taken as any form of trauma to airways such as dental injury, vocal cord injury (manifested as hoarseness within two hours or 48 hours of extubation), or any form of injury to the oral cavity.

### Risk of bias within studies

Two reviewers (PKH, RKY) independently assessed the risk of bias and the quality of the study. Disagreements between the authors was resolved by further discussions. Cochrane risk of bias tool for randomised trial version 2 (ROB 2) [9] was used for the assessment of randomisation process, deviation from intended interventions, missing outcome data, measurement of outcome, selection of reported result, and the overall risk of bias. We also used the risk of bias assessment tool for non-randomised studies (ROBINS-I) [10], which is an assessment tool for qualitative studies. National Institute of health(NIH) tool for observation studies was used for the assessment of bias for observational studies [11].

### Summary measures and synthesis of results

Systematic narrative synthesis of the finding from the included studies was done. Description of populations, intervention, study design, outcome, bias and quality of evidence were summarized. Review Manager, version 5.3.1 was used for a dichotomous outcome (first-pass success, Cormack Lehane grade ≤ 2, Cormack Lehane grade >2, use of external laryngeal maneuver, stylet use, airway injury). Odds ratio (OR) with 95% confidence interval was calculated for the dichotomous variable. For continuous measures (time of intubation) we calculated the mean difference (MD). For multiarmed studies, we analysed data of two arms, one with a customized form of videolaryngoscope and the other with the control arm, where a conventional laryngoscope of any type was used. GRADE (Grading of Recommendation, Assessment, Development and Evaluation) approach will be used to assess the certainty of the evidence for the eligible study.

Decisions on meta-analysis were planned to be made on a consensus regarding the quality of evidence synthesized from the systematic review and whether the recommendation was useful or not. We planned to conduct a meta-analysis for similar outcomes, the fixed effect model or random-effects model was planned to be used based on the nature of heterogeneity. Heterogeneity was proposed to be assessed using I square.

Subgroup analysis was proposed to be performed if the results of the metanalysis differed according to the use of different conventional laryngoscope (for e.g. Macintosh or Millers laryngoscope), different age groups, anticipated of known difficult laryngoscopy, the experience of the operator, obese or non-obese participant and the site of intubation (emergency, operation theatre, intensive care unit).

## Results

### Study selection and characteristics

**Fig 1:**
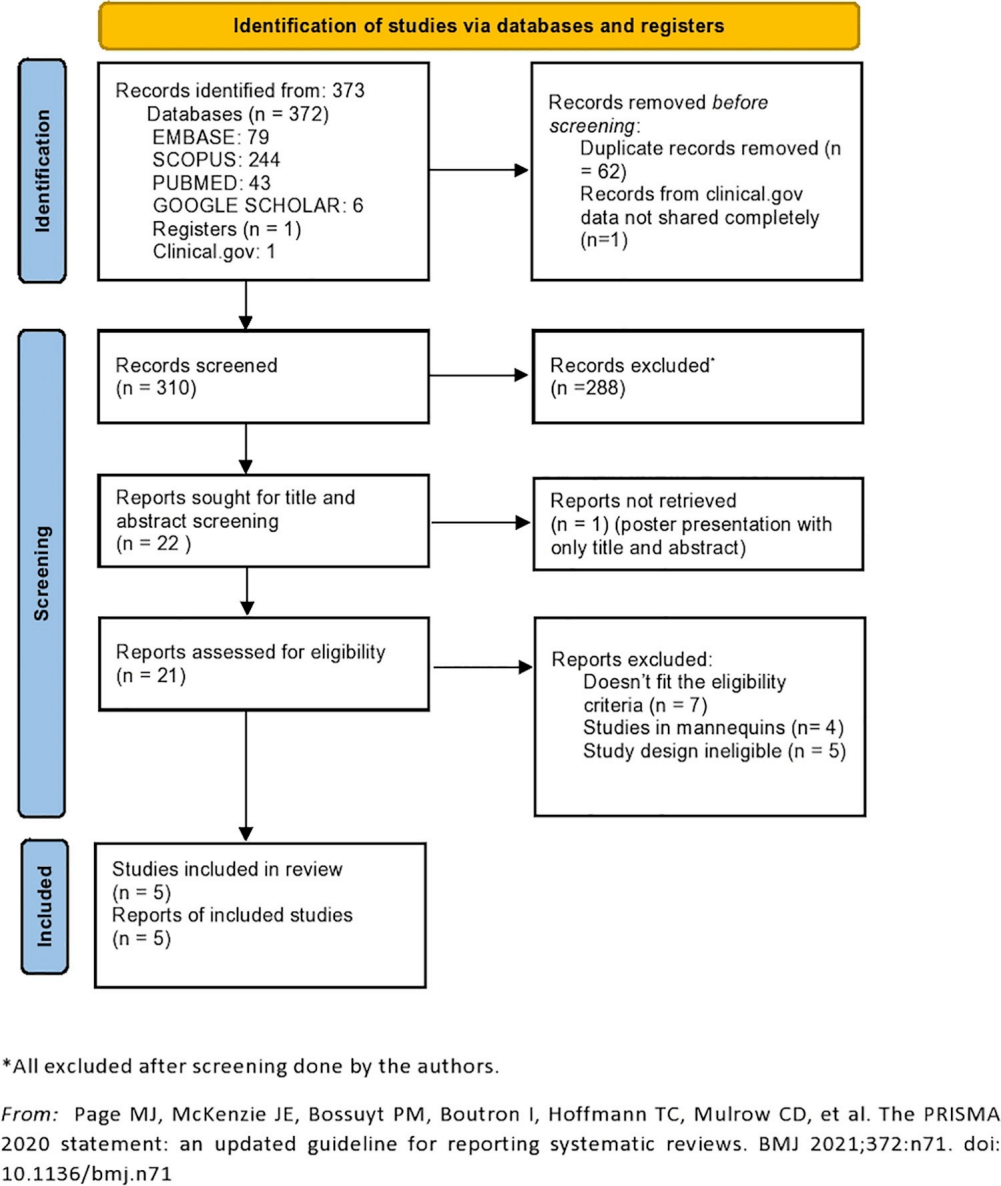
PRISMA flowchart of the included studies.

We identified total of 373 studies from the databases and the registry using the search terms. One of the studies was ongoing and registered in clinicaltrials.gov but did not have the complete data shared as of May 31, 2021. A total of 62 duplicates were removed thereafter, with 310 studies that were screened for title and abstract. Only 22 studies were retrieved after screening for title and abstract. Out of 22 studies, only 21 were assessed for eligibility as one of the studies was a poster presentation at the conference, the details of which were not retrievable [5]. The details of the search strategies in different search engines and databases are outlined in PRISMA flowchart (Fig 1).

Only five studies were used for the final review. Out of 21, 4 studies that were done in manikin also used customised forms of video laryngoscope and were excluded [3, 12–14]. Another 7 studies although had proposed a novel form of videolaryngoscope in the title, were not customised form for use [15–21] Other 5 studies out of 21 were editorials, case series, letters to editors with no human participation involved and with no intervention [4, 22–25]. The remaining five studies were eligible and contained data with intervention performed in the patients [6, 26–29]. Out of five, only three studies had a comparison group [26–28].

### Summary of evidence and risk of bias within included studies

All the studies included were cross-sectional studies. Except for one study [26], other studies were graded as fair to poor quality based on NIH observational tool assessments (Table 2). Almost all studies had the research objective stated and population defined. Participation eligibility was also explained in all studies. Almost all studies except one study [26] had no justification for sample size, power description and effect estimates. Exposures measures were defined clearly in all studies except one [29]. However, the consistency of the exposures variable across all study participants was questionable among all studies. Key potential confounding variables were not clearly adjusted statistically for their impact on the relationship between exposure and outcome in almost all studies. A more detailed summary of evidence and the risk of bias assessment among the included studies is presented (Tables 1 and 2).

**Table 1:** Summary of the finding of all included studies.

Study details, Authors	Karippacheril 2014	Prasanna 2017	Luqman 2017	Ameya 2020	Hernandez 2020	Certainty of evidence using GRADE Approach*
**Country**	India	India	India	India	Mexico	-
**Aim/Objective of the study**	To describe the initial experience using an inexpensive custom-made device that can be used to perform video-laryngoscopy.	Evaluate custom made low-cost straight blade laryngoscope (v-scope) compared to conventional miller blade	Evaluate between custom made video laryngoscope and Macintosh laryngoscope aided endotracheal intubation	Primary objective was to compare and evaluate different airway devices	Comparison of successfully intubating patient with hybrid Videolaryngoscope (VDL Hybrid 1.0)	-
**Study design**	Cross-sectional study	Pilot study	Cross-sectional study	Cross-sectional study	Cross-sectional study	-
**Sampling technique**	simple random sampling (method not described)	systematic random sampling via computer generated method	simple random sampling (lottery method)	Allocation sequence generated using random numbers table and concealed in an envelope	Random allocation to two groups	-
**Population**	Anesthesiology consultant with more than 8 years of experience, Total patients = 24	Trainee Anesthesiologist (first year postgraduate), Total patients = 40, 20 in each group	Experienced anesthesiologist, Total patients: 50 with 25 in each group	Anesthesiologist, Total patients: 60, with 30 in each group	Total 60 = Resident and physician second year [29], Third year physician [22], Base physician [7]. All randomized to two groups of 30 each	-
**Intervention**	Custom made device assembled using a USB endoscopic camera and conventional Macintosh laryngoscope blade size 4 and connected to a Custom device assembled using a waterproof USB endoscopic camera, a conventional Macintosh laryngoscope blade size 3 or 4, a computer.	V-scope (borescope attached to conventional Millers blade using waterproof tapes and connected to a smartphone)	Custom made device assembled using a USB endoscopic camera and conventional Macintosh laryngoscope blade size 4 and connected to a laptop	Videoendoscope consisting of surgical endoscope used in conjunction with Macintosh blade size 3. Endoscope attached to the standard light source and a video camera	Used shovel racket build from medical grade resin with the handle to accommodate the video module and space in the shovel to accommodate its wires connected to light emitting diodes. The whole system was managed with a mobile application allowing the user to view transmitted image	-
**Comparator**	None	Miller’s Laryngoscope	Macintosh laryngoscope	Macintosh Laryngoscope	None	-
**Level of airway difficulty**	No exclusion of difficult airway mentioned	Excluded patient with risk of aspiration, oropharyngeal pathology, ASA grade 3 and 4, restricted neck and mouth movement, cervical instability, Modified Mallampati grade 4, BMI more than 35 kg/m2, Neck Circumference 41 cm (M), 39 cm (F) and history of difficult airway or sleep apnea.	Excluded patient with the history of difficult intubations, anticipated difficulties, and increase risk of pulmonary aspirations	Excluded cases were those with Inter-incisor distance less than 3 cm, Respiratory tract infections, cervical injury, risk of aspirations, included patients with at least one of the difficult airways (history of difficulty previously, Thyromental distance 6 cm, sternomental distance 12 cm, limited neck extension, modified Mallampati grade of 3 or 4)	Difficult airway excluded in preoperative assessment.	-
**First attempt ET intubation success**	Not mentioned	Not mentioned	CL-16/25, CVL-22/25	CL- 29/30, CVL-28/30	Not mentioned	⨁◯◯◯ VERY LOW (2 observation studies)
**Intubation time (seconds)**	CL: 28.58 +/- 21.01	CL: 62.2 +/- 25.1, CVL: 53.1 +/- 24.2	CL: 40.64 +/- 5.70, CVL: 26.92 +/- 5.03	CL: 50.57 +/- 33.74, CVL: 29.73 +/- 11.65	CVL: 23.5 (16–54)	⨁◯◯◯ VERY LOW (3 observational studies)
**Number of attempts (more than 1)**	Not mentioned	Not mentioned	CL-9/25, CVL-3/25	CL- 1/30, CVL-2/30	CVL: 1/30	⨁◯◯◯ VERY LOW (2 observational studies)
**Cormack Lehane grade [grade1/2/3]**	Cl: 9/15/00	CL: 7/9/04, CVL: 16/3/1	CL: 12/11/2, CVL: 15/9/1	Cl: 26/2/2, CVL: 6/18/6	CVL: 28/2/0/0	⨁◯◯◯ VERY LOW (Cormack Lehane ≤ 2 and Cormack Lehane > 2 were separately assessed both yielding the same grade; 3 observational study assessed)
**POGO score (%)**	CVL: 62.29 +/- 28.40	Not mentioned	[100%/50-100%/<50%], CL: 10/11/14, CVL: 13/10/2	Not mentioned	Not mentioned	2 different observational studies, one with a numerical value and the other with ranges, hence not assessed
**External laryngeal manipulation**	8/24	Not mentioned	Not mentioned	Not mentioned	Not mentioned	Only one study hence not assessed
**Stylet use**	All cases used stylet while intubation	Not mentioned	Not mentioned	Not mentioned	Not mentioned	Only one study hence not assessed
**Airway injuries**	Minor blood staining (2/24)	No dental trauma, sore throat or hoarseness noted in both groups	Not mentioned	One case in each reported sore throat following extubation	Not mentioned	Variable data to assess.
**Level of evidence as per NIH protocol**	Poor	Fair	Fair	Good	Fair	

CL: Conventional Laryngoscope, CVL: Custom Videolaryngoscope, ET: Endotracheal, POGO: Percentage of glottic opening, NIH: National Institute of Health, USB: Universal Serial Bus.

*Certainty of evidence is graded using GRADE (Grading of Recommendation, Assessment, Development and Evaluation) approach using Gradepro program by Cochrane Collaboration.

**Table 2:** Risk of bias assessment according to National Institute of Health quality assessment tool for cross-sectional study.

List of items	Prasanna 2017	Luqman 2017	Karipachheril 2014	Ameya 2020	Hernandez 2020
1. Was the research question or objective in this paper clearly stated?	yes	yes	yes	yes	yes
2. Was the study population clearly specified and defined?	yes	yes	yes	yes	yes
3. Was the participation rate of eligible persons at least 50%?	yes	yes	yes	yes	yes
4. Were all the subjects selected or recruited from the same or similar populations (including the same time period)? Were inclusion and exclusion criteria for being in the study prespecified and applied uniformly to all participants?	yes	yes	yes	yes	no
5. Was a sample size justification, power description, or variance and effect estimates provided?	no	no	no	yes	no
6. For the analyses in this paper, were the exposure(s) of interest measured prior to the outcome(s) being measured?	no	no	no	yes	no
7. Was the timeframe sufficient so that one could reasonably expect to see an association between exposure and outcome if it existed?	no	no	no	yes	no
8. For exposures that can vary in amount or level, did the study examine different levels of the exposure as related to the outcome (e.g., categories of exposure, or exposure measured as continuous variable)?	no	no	no	Yes	no
9. Were the exposure measures (independent variables) clearly defined, valid, reliable, and implemented consistently across all study participants?	yes	no	yes	yes	no
10. Was the exposure(s) assessed more than once over time?	no	no	no	no	no
11. Were the outcome measures (dependent variables) clearly defined, valid, reliable, and implemented consistently across all study participants?	yes	no	no	yes	yes
12. Were the outcome assessors blinded to the exposure status of participants?	no	no	no	no	no
13. Was loss to follow-up after baseline 20% or less?	no	no	no	no	
14. Were key potential confounding variables measured and adjusted statistically for their impact on the relationship between exposure(s) and outcome(s)?	yes	no	no	yes	yes
**Quality Rating**	**FAIR**	**FAIR**	**POOR**	**GOOD**	**FAIR**

### Synthesis of results

#### Primary outcomes

First tracheal intubation attempt success was mentioned by only three studies [6, 26, 27] Only two studies [26, 27] had the comparison group.

#### Secondary outcomes

*Intubation time*. Out of 5 studies, only 3 [26–28] had the intervention group. All studies explained insertion of videolaryngoscope or laryngoscope insertion in the oral cavity as the initial time.

*Number of attempts*. Three studies [26, 27, 29] mentioned the number of attempts more than one, however only two [26, 27] had the comparison group.

*Laryngoscopic view*. Out of 5 studies, Cormack Lehane (CL) grade was compared in 3 studies with conventional laryngoscope and custom videolaryngoscope group. Two studies mentioned POGO Score, where one study had the comparison group [27] and other one studies had no comparison group [6]. Although, we planned to group CL grade into two groups for meta-analysis: CL grade >2 and CL grade ≤ 2, no further analysis was done due to poor quality of evidence.

*External laryngeal manipulation (ELM) and use of stylet*. Only 2 studies [6, 28] out of 5 mentioned using ELM for aiding in the laryngoscopic view and successful tracheal intubation. No studies with a control arm had interpreted ELM. One study mentioned stylet use for endotracheal intubation [6] where stylet was used for all the cases, however, the study did not have the conventional laryngoscope group to compare.

*Airway injury*. Three studies [6, 26, 28] reported some form of the airway injury. Two studies which had used the conventional laryngoscope as control didn’t mention any form of airway injury described as dental, oral injury or the hoarseness of voice. Only one study with custom videolaryngoscope reported 2 minor blood stains out of total of 24 cases after the endotracheal intubation [6].

### Meta-analysis

The quality of evidence for five studies was from fair to poor and hence it was decided together by all three authors that the meta-analysis will not guide the generation of useful good evidence. For the meta-analysis, there were 2 studies with conventional laryngoscope as a comparison group for first attempt success, number of attempts for successful tracheal intubation. Similarly, 3 studies had a comparison group for intubation time, Cormack Lehane with different grades.

## Discussions

Standard videolaryngoscope is largely accessible in developed countries and regarded as the standard of care during tracheal intubation, however, developing countries are still struggling to acquire it for regular use reasons mainly related to cost issues [30]. Conventional laryngoscope has been modified for use of low cost teaching aids [14] and proposed for use at the time of health disasters [31]. Various modifications were proposed in low resourced settings however, the performance of these non-commercial devices has not been aggregated and evaluated to provide an evidence-based recommendation for use. Most systematic reviews and the meta-analysis with videolaryngoscope take into consideration the use of standard form of videolaryngoscope however, it is yet to be determined whether the customised form of this videolaryngoscope are efficacious and safe for the patients even when used as an improvised form in low- and middle-income countries.

### Summary of main results

Out of total of 5 studies, we found only 3 observation studies where custom videolaryngoscope was compared with a conventional laryngoscope. Out of the 3, two studies used Macintosh laryngoscope of size three and four [26, 27] and one study used Miller’s Blade [28]. The remaining two studies did not have the comparison group. The modifications on the laryngoscope device was different for all 5 studies. Prasanna et al. used V-scope (borescope attached to conventional Millers blade using waterproof tapes and connected to a smartphone) [28]. Luqman et al. used a custom made device assembled using a USB endoscopic camera and conventional Macintosh laryngoscope blade size four and connected to a laptop [27]. Karipachheril et al. used a Custom made device assembled using an USB endoscopic camera and a conventional Macintosh laryngoscope blade size 4 and connected to a Custom device assembled using a waterproof USB endoscopic camera, a conventional Macintosh laryngoscope blade size 3 or 4, and a computer [6]. Ameya et al. reported a slightly different technique as they used video endoscope consisting of a surgical endoscope used in conjunction with the Macintosh blade size 3, an endoscope attached to standard light source and a video camera [26]. Mauricio Hernandez et al. used a shovel racket built from medical grade resin with a handle to accommodate the video module and space in the shovel to accommodate its wires connected to a light emitting diodes. The whole system was managed with a mobile application allowing the user to view transmitted image [29].

Only one study as per NIH tool assessment for observational studies was graded as good [26]. One study was very poor as it didn’t take any consideration on sample size calculation with power description and, effect estimates. It had a poor selection of candidates, lack of blinding of assessors and had confounding variables that would affect the outcome [6]. In 2 studies there was no clarity in confounding variables [27, 29]. All 3 studies [27–29] with fair grades had taken no consideration in sample size, power description, or provided effect estimates. All studies suggested different levels of difficult airway characteristics that would have affected the outcome. All 5 studies included elective surgery patients undergoing general anesthesia. Three studies excluded different causes of anticipated difficult airway, previous history of difficult airway and those at risk of pulmonary aspiration. In contrast, one study [26] included patients who had at least one difficult airway characteristics (history of difficulty previously, Thyromental distance ≤ 6 cm, sternomental distance ≥ 12 cm, limited neck extension, modified Mallampati grade of 3 or 4. One study didn’t mention any level of difficulty in airway with an only random selection of elective surgery patients [6]. Details of the summary of finding is enlisted in Table 1.

Almost all four studies when assessed with GRADE approach were found to be “Very low” in the certainty of evidence. Most studies didn’t have clarity of randomisation process, blinding, participant allocation with a tendency for selective reporting which was graded as a very serious grade of risk of bias assessment. When data are pooled, they looked heterogenous with the tendency to differ in interventions as the modification of videolaryngoscope were different, done in different populations and age groups with no clarity of the training and experience level of the operator (Table 1). Most studies were imprecise with a small sample size and wide confidence interval (Table 1).

### First attempt endotracheal intubation success

The first attempt success for endotracheal intubation was only analysed using a comparison group in two studies [26, 27] both of which were graded as good and fair on the risk of bias assessments respectively. In both studies, attempts were done by an anaesthesiologist with no demarcation on the experience level. However, when the GRADE approach was used the risk were graded as “very low” in the certainty of evidence (Table 1) as there were non-randomised, with no accounts taken on confounding variables with the lack of sample size basis. Data were highly heterogeneous with the wider confidence interval when pooled with great variability in the experience of the person performing endotracheal intubation with some effect arising due to some differences in the type of improvisation in the videolaryngoscope.

### Number of attempts of endotracheal intubation

Only two studies [26, 27] mentioned the number of attempts with comparison to conventional laryngoscope. Even though one of the study mentioned number of attempts it did not have a comparison group [6]. Using GRADE approach, the certainty of evidence was “very low” because data has a serious risk of bias, inconsistent, imprecise with no directions.

### Endotracheal intubation time

Three studies [26–28] were analysed for intubation time in two groups. All 3 studies mentioned the insertion of the videolaryngoscope in the oral cavity as the starting point. For final endpoint, two study [26, 28] mentioned end tidal capnography trace for successful insertion and intubation time. Remaining, one study took the direct view of endotracheal tube insertion as final point [27]. It was worth noting that in one study [28] the operator probably took a longer time for intubation as it was found to be done by first year postgraduate trainee anesthesiologist. The other two studies [26, 27] was done by anesthesiologist with no definite level of experience defined. Although the data were more consistent in the three studies, they were non-randomized with confounding variables that could affect the outcome leading to “very low” grading in the GRADE approach.

### Laryngoscopic view

Laryngoscopic view was planned to be assessed as ≤ 2 and > 2 CL grade for meta-analysis but it was not performed as the studies were poorly graded on a risk of bias assessments [26–28]. All three studies mentioned Grade 1, 2 and 3 however no study mentioned Grade 4 of CL. The finding was based upon the intubator view at the time of laryngoscopy. However, in both cases, they had the serious risk of bias as the studies were non-randomized, with confounding variables not taken in consideration. The data were inconsistent, lacking direction. Data were also imprecise as the population were variable with shuttle variation in the customization of the improvised laryngoscope with fewer sample with the wide confidence interval to make a conclusion (Table 1).

### External aid

External aid in the form of external laryngeal manipulation (ELM) to improve the laryngoscopic view was not mentioned in any study which comprised of a comparison group. Only one study had mentioned the use of ELM for improving the intubating condition and laryngoscopic view [6]. In the same study, stylet was used in all cases that would have bearing on the first attempt success, number of attempts and intubation time.

### Airway injury

Only one study with comparison group analysed airway injury in the form of dental trauma, sore throat and hoarseness with lack of details on the follow up post-surgery for any adverse events [28]. However no cases were found to have incidences of airway injury. Another study with no comparison group reported two cases out of 24 with a minor injury in the videolaryngoscope after intubation [6].

### Limitations of the systematic review

We didn’t do the meta-analysis because none of the outcome of the studies which were graded fair to poor as per the NIH tool assessment seemed to guide in generation of good evidence. It is difficult to assure the actual level of training of the intubator who performed endotracheal intubation with customised videolaryngoscope or conventional laryngoscope. It was difficult to differentiate level of difficulty in airway in most of the studies. Use of customised videolaryngoscope had not been explored in the emergency settings, in critical care unit or in the emergency situations. It was also difficult to comment on the variable performances of the different customised designs. There is a possibility that new additional research after our last search date 31^st^ May 2021 may come up with a more concrete result. More incorporation of the data and updates of new devices from the new studies may lead to the changes of the result of this review. There is also the great uncertainty regarding the quality of evidence as most were observational studies with small numbers with fair to low risk of bias, hence high quality randomised controlled trials among the different customized videolaryngoscope can pave the way with more clarity on certainty for the evidence. Airway injury using this device is one of the most important outcomes which seems to be seldom analysed while designing these studies.

To conclude, the certainty of evidence regarding the performance of custom videolaryngoscope compared to conventional laryngoscope is very low, done in small numbers with fair to poor risk of bias. The data are mostly inconsistent, raising issues of the applicability. It is difficult to establish and do further analysis on whether the customised forms of videolaryngoscopes will improve the first attempt successful tracheal intubation, reduce the number of attempts, improve laryngoscopic view, require fewer external aids and reduce the incidences of airway injury. Well-designed randomised trials in good numbers and adequate samples will be required to further analyze the outcome and make the strong recommendation as the existing evidence are very poor at this time of analysis.

## Supporting information

S1 Table PRISMA guideline checklist. (TIFF)

S2 Table Search strategies of the included studies. (TIFF)

S3 Table AMSTAR-2 rating [32]. (TIFF)
