# Supplementary figures and images for: Performance of custom made videolaryngoscope for endotracheal intubation: A systematic review
Source: PLoS One. 2022 Jan 6;17(1):e0261863. doi: 10.1371/journal.pone.0261863 (PMC8735609; doi:10.1371/journal.pone.0261863)

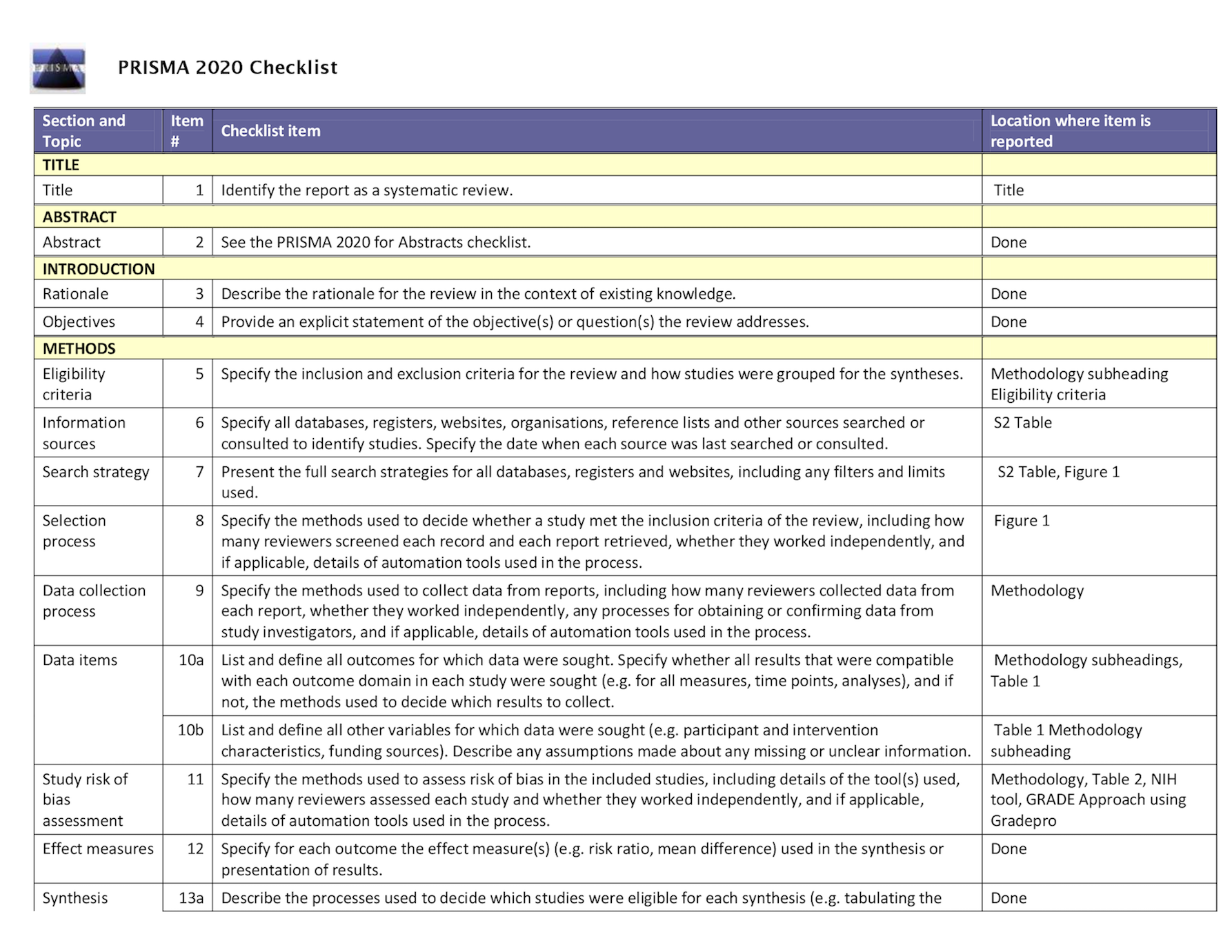

Supplement: S1 Table — (TIFF) [file pone.0261863.s001.tiff]

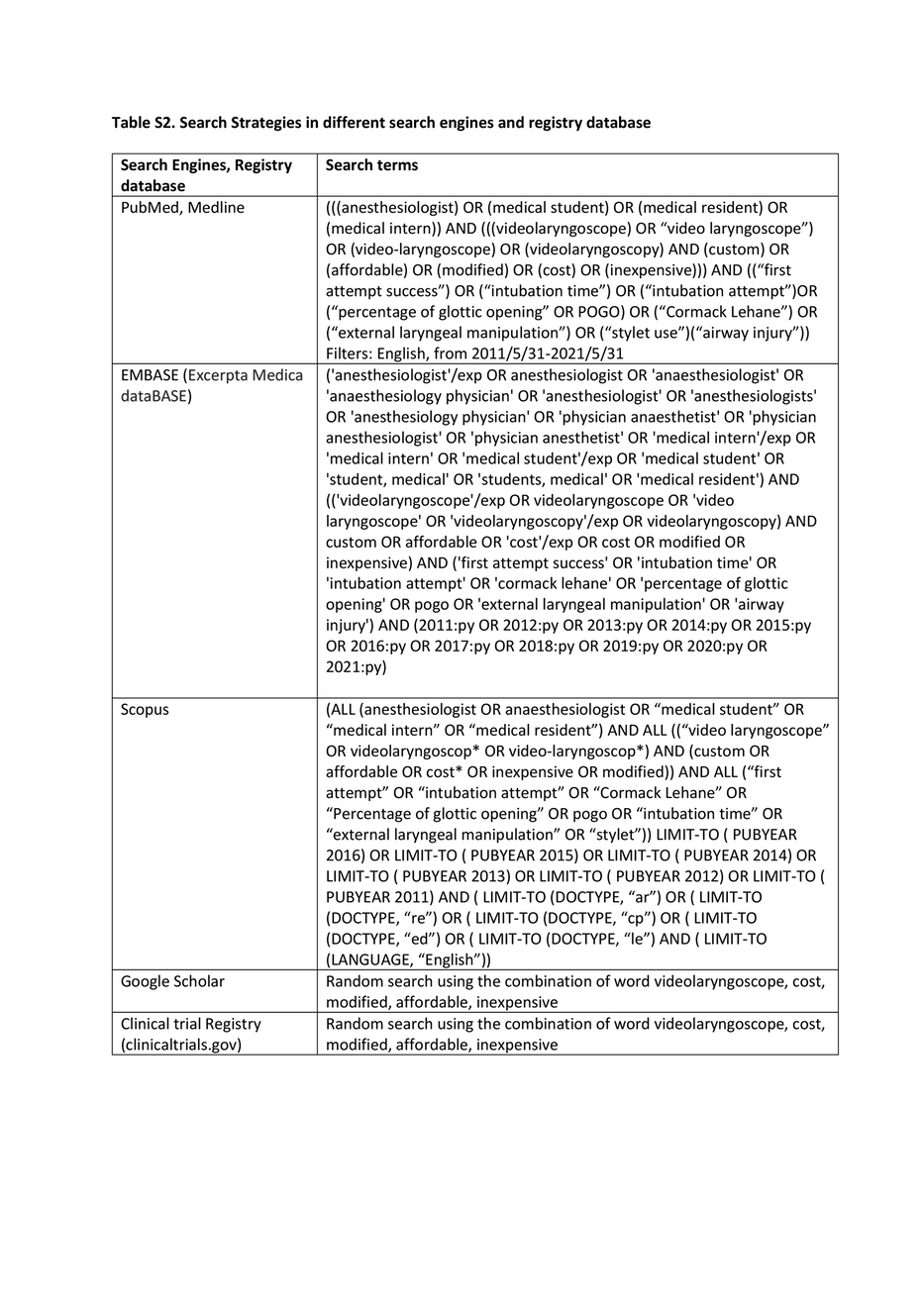

Supplement: S2 Table — (TIFF) [file pone.0261863.s002.tiff]

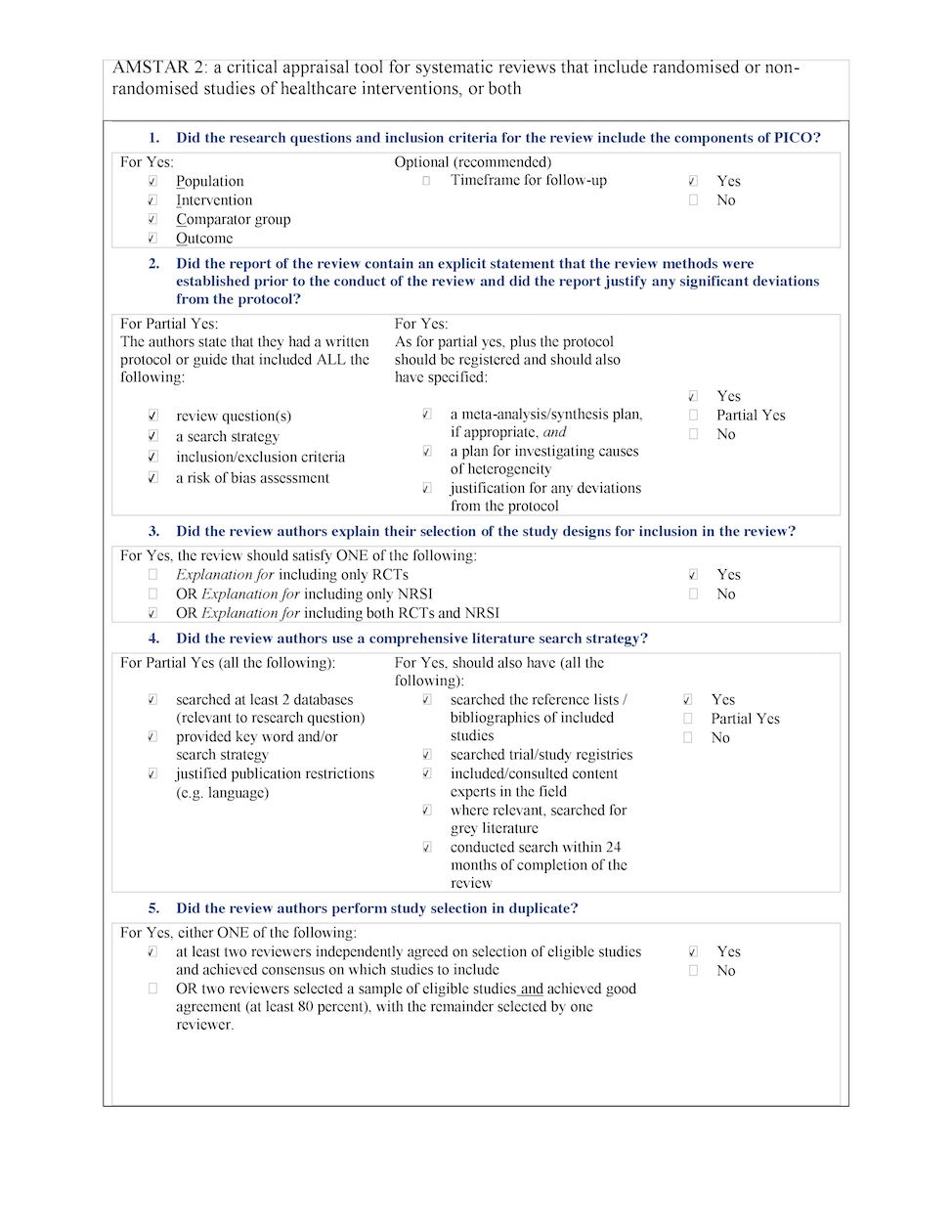

Supplement: S3 Table — (TIFF) [file pone.0261863.s003.tiff]
